# 2,2′-(Disulfanedi­yl)dianilinium dichloride dihydrate

**DOI:** 10.1107/S1600536813015742

**Published:** 2013-06-12

**Authors:** Hasna Bouchareb, Mhamed Boudraa, Sofiane Bouacida, Hocin Merazig

**Affiliations:** a Unité de Recherche de Chimie de l’Environnement et Moléculaire Structurale, CHEMS, Université Mentouri-Constantine, 25000, Algeria; b Département Sciences de la Matière, Faculté des Sciences Exactes et Sciences de la Nature et de la Vie, Université Oum El Bouaghi, Algeria

## Abstract

In the title hydrated mol­ecular salt, C_12_H_14_N_2_S_2_^2+^·2Cl^−^·2H_2_O, the dihedral angle between the benzene rings in the dication is 9.03 (17)° and the C—S—S—C torsion angle is 96.8 (2)°. The crystal packing can be described as alternating organic and anionic water layers lying parallel to (100), which are linked by N—H⋯Cl and N—H⋯O hydrogen bonds. O—H⋯Cl hydrogen bonds and aromatic π–π stacking inter­actions [centroid–centroid separation = 3.730 (3) Å] are also observed.

## Related literature

For related structures and background to di­sulfides, see: Benmebarek *et al.* (2012 ▶, 2013 ▶). For related structures, see: Tang *et al.* (2011 ▶); Goh *et al.* (2010 ▶); Song & Fan (2009 ▶).
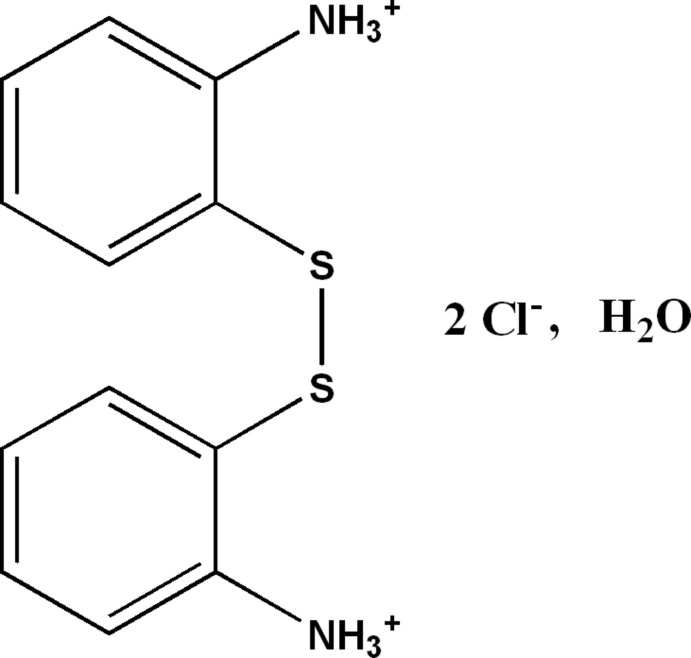


## Experimental

### 

#### Crystal data

C_12_H_14_N_2_S_2_^2+^·2Cl^−^·2H_2_O
*M* *_r_* = 357.32
Orthorhombic, 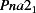

*a* = 17.826 (7) Å
*b* = 13.358 (5) Å
*c* = 7.120 (3) Å
*V* = 1695.4 (12) Å^3^
*Z* = 4
Mo *K*α radiation
μ = 0.63 mm^−1^
*T* = 150 K
0.16 × 0.13 × 0.11 mm

#### Data collection

Bruker APEXII CCD diffractometer
10760 measured reflections
3584 independent reflections
2409 reflections with *I* > 2σ(*I*)
*R*_int_ = 0.097

#### Refinement

*R*[*F* ^2^ > 2σ(*F* ^2^)] = 0.063
*wR*(*F* ^2^) = 0.134
*S* = 1.05
3584 reflections
195 parameters
6 restraints
H atoms treated by a mixture of independent and constrained refinement
Δρ_max_ = 0.58 e Å^−3^
Δρ_min_ = −0.47 e Å^−3^
Absolute structure: Flack (1983 ▶), 1369 Friedel pairs
Flack parameter: −0.12 (12)

### 

Data collection: *APEX2* (Bruker, 2011 ▶); cell refinement: *SAINT* (Bruker, 2011 ▶); data reduction: *SAINT*; program(s) used to solve structure: *SIR2002* (Burla *et al.*, 2005 ▶); program(s) used to refine structure: *SHELXL97* (Sheldrick, 2008 ▶); molecular graphics: *ORTEP-3 for Windows* (Farrugia, 2012 ▶) and *DIAMOND* (Brandenburg & Berndt, 2001 ▶); software used to prepare material for publication: *WinGX* (Farrugia, 2012 ▶).

## Supplementary Material

Crystal structure: contains datablock(s) global, I. DOI: 10.1107/S1600536813015742/hb7089sup1.cif

Structure factors: contains datablock(s) I. DOI: 10.1107/S1600536813015742/hb7089Isup2.hkl

Supplementary material file. DOI: 10.1107/S1600536813015742/hb7089Isup3.cml

Additional supplementary materials:  crystallographic information; 3D view; checkCIF report

## Figures and Tables

**Table 1:** Hydrogen-bond geometry (Å, °)

*D*—H⋯*A*	*D*—H	H⋯*A*	*D*⋯*A*	*D*—H⋯*A*
N1—H1*A*⋯O1*W* ^i^	0.89	1.83	2.723 (6)	178
N1—H1*B*⋯Cl2^i^	0.89	2.24	3.108 (4)	166
N1—H1*C*⋯Cl1^i^	0.89	2.25	3.103 (4)	160
N2—H2*A*⋯O2*W* ^ii^	0.89	1.84	2.727 (6)	177
N2—H2*B*⋯Cl2^iii^	0.89	2.26	3.111 (4)	160
N2—H2*C*⋯Cl2	0.89	2.30	3.157 (4)	163
O2*W*—H4*W*⋯Cl2	0.86 (5)	2.36 (5)	3.157 (5)	155 (5)
O2*W*—H3*W*⋯Cl1^iv^	0.85 (5)	2.23 (5)	3.078 (4)	171 (6)
O1*W*—H1*W*⋯Cl1^v^	0.85 (5)	2.27 (5)	3.096 (5)	167 (5)
O1*W*—H2*W*⋯Cl1^iv^	0.86 (5)	2.27 (5)	3.127 (5)	176 (7)

Symmetry codes: (i) 
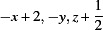
; (ii) 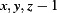; (iii) 
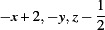
; (iv) 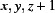; (v) 
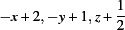
.

**Fig. 1.**
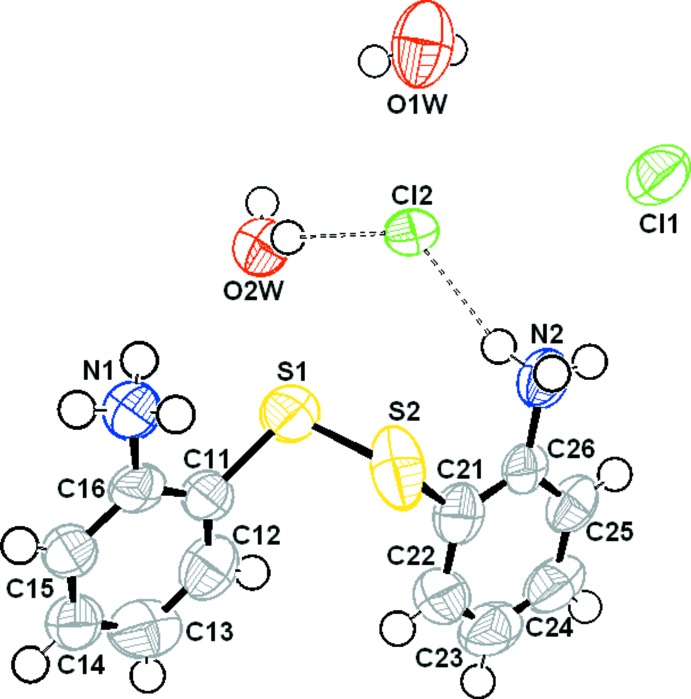
The molecular structure of (I) with displacement ellipsoids drawn at the 50% probability level.

**Fig. 2.**
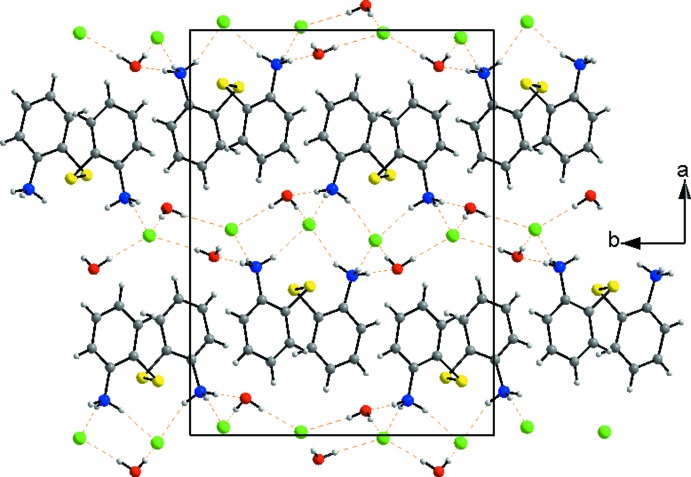
Diagram packing of (I) viwed via c axis showing hydrogen bonding in alterning layers.

## supplementary crystallographic information

### Comment

As part of our ongoing studies on the synthesis, structures and biological activity of organometallic complexes based in sulfur (Benmebarek *et al.* 2012 and Benmebarek *et al.* 2013), we have synthesized and determined the crystal structure of the title compound (I), (Fig. 1). In the cation the S—S bond length is 2.061 (2)°, indicating the single bond character similar to that found in 4,4'-diaminophenyldisulfide (Tang *et al.*, 2011; Goh *et al.* 2010). In the diprotoned 2,2'-dithiodianiline moiety, the dihedral angle between the benzene rings is 9.03 (17)°; different to that found in [67.82 (9)°] 1,2-Bis(2-nitrophenyl)disulfane (Song & Fan, 2009) and [39.9 (2)°]4,4'- diaminophenyldisulfide (Tang *et al.*, 2011). The crystal packing can be described as alternating layers parallel to (100) plane, wich are linked toghether by N—H···Cl and N—H···O interactions involving molecule of water and anions chloride. O—H···Cl hydrogen bond and π–π stacking are observed.

### Experimental

2-Aminobenzenethiol (0.1 mmol) was added to concentrated HCl (2 ml) and transfered into a 23 ml teflon-lined stainless steel autoclave and heated at 120° C for 3 days. Then the autoclave was cooled to room temperature at 10°/h. Colourless prisms were collected, washed with ethanol and dried in air at room temperature.

### Refinement

Approximate positions for all H atoms were first obtained from the difference electron density map. However, the H atoms were situated into idealized positions and the H-atoms have been refined within the riding atom approximation. The applied constraints were as follow: C_aryl_—H_aryl_ = 0.93 Å and N_ammonium_—H_ammonium_ = 0.89 Å. *U*_iso_(H_aryl_) = 1.2*U*_eq_(C_aryl_). *U*_iso_(H_ammonium_) = 1.5*U*_eq_(C_ammonium_). Except for H1W, H2W, H3W and H4W (of water molecule) were located in a difference Fourier map and refined isotropically with *U*_iso_(H) = 1.5*U*_eq_(O).

### Crystal data

**Table:**

C_12_H_14_N_2_S_2_^2+^·2Cl^−^·2H_2_O	*F*(000) = 744
*M**_r_* = 357.32	*D*_x_ = 1.4 Mg m^−^^3^
Orthorhombic, *P**n**a*2_1_	Mo *K*α radiation, λ = 0.71073 Å
Hall symbol: P 2c -2n	Cell parameters from 2698 reflections
*a* = 17.826 (7) Å	θ = 2.3–28.5°
*b* = 13.358 (5) Å	µ = 0.63 mm^−^^1^
*c* = 7.120 (3) Å	*T* = 150 K
*V* = 1695.4 (12) Å^3^	Prism, colourless
*Z* = 4	0.16 × 0.13 × 0.11 mm

### Data collection

**Table:**

Bruker APEXII CCD diffractometer	2409 reflections with *I* > 2σ(*I*)
Radiation source: sealed tube	*R*_int_ = 0.097
Graphite monochromator	θ_max_ = 28.8°, θ_min_ = 2.8°
φ and ω scans	*h* = −24→23
10760 measured reflections	*k* = −18→18
3584 independent reflections	*l* = −9→8

### Refinement

**Table:**

Refinement on *F*^2^	Secondary atom site location: difference Fourier map
Least-squares matrix: full	Hydrogen site location: inferred from neighbouring sites
*R*[*F*^2^ > 2σ(*F*^2^)] = 0.063	H atoms treated by a mixture of independent and constrained refinement
*wR*(*F*^2^) = 0.134	*w* = 1/[σ^2^(*F*_o_^2^) + (0.0595*P*)^2^] where *P* = (*F*_o_^2^ + 2*F*_c_^2^)/3
*S* = 1.05	(Δ/σ)_max_ < 0.001
3584 reflections	Δρ_max_ = 0.58 e Å^−^^3^
195 parameters	Δρ_min_ = −0.47 e Å^−^^3^
6 restraints	Absolute structure: Flack (1983), 1369 Friedel pairs
Primary atom site location: structure-invariant direct methods	Flack parameter: −0.12 (12)

### Special details

**Table:**

Geometry. All e.s.d.'s (except the e.s.d. in the dihedral angle between two l.s. planes) are estimated using the full covariance matrix. The cell e.s.d.'s are taken into account individually in the estimation of e.s.d.'s in distances, angles and torsion angles; correlations between e.s.d.'s in cell parameters are only used when they are defined by crystal symmetry. An approximate (isotropic) treatment of cell e.s.d.'s is used for estimating e.s.d.'s involving l.s. planes.
Refinement. Refinement of *F*^2^ against ALL reflections. The weighted *R*-factor *wR* and goodness of fit *S* are based on *F*^2^, conventional *R*-factors *R* are based on *F*, with *F* set to zero for negative *F*^2^. The threshold expression of *F*^2^ > σ(*F*^2^) is used only for calculating *R*-factors(gt) *etc*. and is not relevant to the choice of reflections for refinement. *R*-factors based on *F*^2^ are statistically about twice as large as those based on *F*, and *R*- factors based on ALL data will be even larger.

### Fractional atomic coordinates and isotropic or equivalent isotropic displacement parameters (Å^2^)

**Table:**

	*x*	*y*	*z*	*U*_iso_*/*U*_eq_	
Cl2	0.98060 (6)	0.10914 (8)	1.19390 (17)	0.0269 (3)	
S1	0.86675 (9)	−0.10667 (11)	1.3286 (2)	0.0412 (4)	
Cl1	0.99242 (8)	0.36455 (10)	0.7389 (2)	0.0411 (4)	
S2	0.85864 (8)	−0.15845 (11)	1.0567 (2)	0.0395 (4)	
O2W	0.9096 (2)	0.1819 (3)	1.5764 (6)	0.0381 (10)	
H3W	0.935 (3)	0.233 (3)	1.609 (9)	0.057*	
H4W	0.937 (3)	0.150 (4)	1.497 (7)	0.057*	
N2	0.8933 (2)	0.0331 (3)	0.8354 (6)	0.0267 (9)	
H2A	0.8991	0.0831	0.7542	0.04*	
H2B	0.9208	−0.019	0.7989	0.04*	
H2C	0.9082	0.0528	0.9489	0.04*	
C21	0.7911 (3)	−0.0781 (4)	0.9473 (8)	0.0286 (12)	
O1W	1.0599 (3)	0.4223 (3)	1.3483 (6)	0.0534 (12)	
H1W	1.043 (4)	0.476 (3)	1.302 (9)	0.08*	
H2W	1.039 (4)	0.406 (4)	1.453 (6)	0.08*	
N1	0.9146 (2)	−0.2839 (3)	1.5749 (6)	0.0282 (10)	
H1A	0.9242	−0.3284	1.6647	0.042*	
H1B	0.9388	−0.2271	1.5996	0.042*	
H1C	0.9298	−0.3079	1.4647	0.042*	
C26	0.8140 (2)	0.0039 (3)	0.8427 (7)	0.0218 (10)	
C14	0.7102 (3)	−0.3103 (4)	1.6647 (8)	0.0317 (12)	
H14	0.6782	−0.3509	1.7342	0.038*	
C12	0.7275 (3)	−0.1730 (4)	1.4523 (8)	0.0332 (13)	
H12	0.7073	−0.1212	1.3811	0.04*	
C25	0.7627 (3)	0.0625 (3)	0.7478 (8)	0.0272 (11)	
H25	0.7787	0.1168	0.6768	0.033*	
C15	0.7858 (3)	−0.3249 (3)	1.6726 (7)	0.0268 (11)	
H15	0.8055	−0.3753	1.7482	0.032*	
C11	0.8050 (3)	−0.1876 (3)	1.4566 (7)	0.0255 (11)	
C23	0.6638 (3)	−0.0391 (4)	0.8641 (8)	0.0395 (14)	
H23	0.6128	−0.0526	0.8741	0.047*	
C24	0.6876 (3)	0.0396 (4)	0.7590 (8)	0.0345 (12)	
H24	0.6528	0.0784	0.6943	0.041*	
C16	0.8335 (3)	−0.2645 (3)	1.5676 (7)	0.0237 (10)	
C13	0.6811 (3)	−0.2350 (4)	1.5534 (8)	0.0381 (13)	
H13	0.6294	−0.2262	1.5468	0.046*	
C22	0.7156 (3)	−0.1002 (4)	0.9572 (8)	0.0354 (13)	
H22	0.6992	−0.1554	1.0255	0.043*	

### Atomic displacement parameters (Å^2^)

**Table:**

	*U*^11^	*U*^22^	*U*^33^	*U*^12^	*U*^13^	*U*^23^
Cl2	0.0198 (5)	0.0332 (6)	0.0276 (7)	−0.0025 (5)	−0.0034 (6)	0.0026 (5)
S1	0.0445 (9)	0.0428 (7)	0.0364 (8)	−0.0186 (7)	−0.0119 (8)	0.0133 (6)
Cl1	0.0422 (8)	0.0424 (7)	0.0388 (9)	−0.0023 (5)	−0.0125 (7)	0.0027 (6)
S2	0.0429 (8)	0.0445 (7)	0.0310 (8)	0.0133 (6)	0.0111 (8)	0.0111 (6)
O2W	0.040 (2)	0.043 (2)	0.031 (3)	−0.0078 (17)	−0.002 (2)	0.0002 (18)
N2	0.029 (2)	0.030 (2)	0.021 (2)	0.0051 (16)	−0.001 (2)	−0.0027 (17)
C21	0.034 (3)	0.032 (3)	0.019 (3)	0.001 (2)	0.004 (3)	−0.007 (2)
O1W	0.072 (3)	0.051 (3)	0.038 (3)	0.019 (2)	0.003 (3)	−0.003 (2)
N1	0.030 (2)	0.030 (2)	0.025 (3)	−0.0031 (17)	0.000 (2)	−0.0016 (18)
C26	0.022 (2)	0.028 (2)	0.015 (3)	0.0005 (18)	0.003 (2)	−0.0089 (19)
C14	0.028 (3)	0.044 (3)	0.023 (3)	−0.007 (2)	0.002 (3)	−0.004 (2)
C12	0.038 (3)	0.035 (3)	0.027 (3)	0.004 (2)	−0.002 (3)	−0.006 (2)
C25	0.030 (3)	0.030 (2)	0.021 (3)	0.005 (2)	−0.003 (3)	−0.006 (2)
C15	0.033 (3)	0.026 (2)	0.021 (3)	−0.0076 (19)	0.000 (3)	−0.0043 (19)
C11	0.030 (3)	0.028 (2)	0.019 (3)	−0.008 (2)	0.002 (2)	−0.006 (2)
C23	0.026 (3)	0.062 (4)	0.030 (4)	−0.006 (3)	0.002 (3)	−0.015 (3)
C24	0.027 (3)	0.046 (3)	0.030 (3)	0.005 (2)	−0.008 (3)	−0.008 (2)
C16	0.024 (2)	0.027 (2)	0.020 (3)	−0.0046 (18)	0.001 (2)	−0.010 (2)
C13	0.024 (3)	0.052 (3)	0.038 (4)	0.000 (2)	0.009 (3)	−0.017 (3)
C22	0.030 (3)	0.049 (3)	0.028 (3)	−0.008 (3)	0.005 (3)	−0.003 (2)

### Geometric parameters (Å, º)

**Table:**

S1—C11	1.792 (5)	C14—C15	1.364 (6)
S1—S2	2.061 (2)	C14—C13	1.380 (8)
S2—C21	1.791 (5)	C14—H14	0.93
O2W—H3W	0.86 (2)	C12—C13	1.375 (8)
O2W—H4W	0.86 (2)	C12—C11	1.396 (7)
N2—C26	1.468 (6)	C12—H12	0.93
N2—H2A	0.89	C25—C24	1.375 (7)
N2—H2B	0.89	C25—H25	0.93
N2—H2C	0.89	C15—C16	1.391 (7)
C21—C22	1.380 (7)	C15—H15	0.93
C21—C26	1.386 (7)	C11—C16	1.391 (7)
O1W—H1W	0.852 (19)	C23—C24	1.358 (8)
O1W—H2W	0.858 (19)	C23—C22	1.399 (8)
N1—C16	1.469 (6)	C23—H23	0.93
N1—H1A	0.89	C24—H24	0.93
N1—H1B	0.89	C13—H13	0.93
N1—H1C	0.89	C22—H22	0.93
C26—C25	1.380 (7)		
			
C11—S1—S2	103.43 (17)	C13—C12—H12	120
C21—S2—S1	104.74 (18)	C11—C12—H12	120
H3W—O2W—H4W	106 (6)	C24—C25—C26	119.4 (5)
C26—N2—H2A	109.5	C24—C25—H25	120.3
C26—N2—H2B	109.5	C26—C25—H25	120.3
H2A—N2—H2B	109.5	C14—C15—C16	119.9 (5)
C26—N2—H2C	109.5	C14—C15—H15	120
H2A—N2—H2C	109.5	C16—C15—H15	120
H2B—N2—H2C	109.5	C16—C11—C12	118.5 (4)
C22—C21—C26	118.9 (5)	C16—C11—S1	120.7 (4)
C22—C21—S2	120.3 (4)	C12—C11—S1	120.8 (4)
C26—C21—S2	120.6 (4)	C24—C23—C22	120.4 (5)
H1W—O1W—H2W	114 (3)	C24—C23—H23	119.8
C16—N1—H1A	109.5	C22—C23—H23	119.8
C16—N1—H1B	109.5	C23—C24—C25	120.5 (5)
H1A—N1—H1B	109.5	C23—C24—H24	119.7
C16—N1—H1C	109.5	C25—C24—H24	119.7
H1A—N1—H1C	109.5	C15—C16—C11	120.7 (4)
H1B—N1—H1C	109.5	C15—C16—N1	118.7 (4)
C25—C26—C21	121.0 (4)	C11—C16—N1	120.6 (4)
C25—C26—N2	118.1 (4)	C12—C13—C14	120.9 (5)
C21—C26—N2	120.8 (4)	C12—C13—H13	119.5
C15—C14—C13	120.0 (5)	C14—C13—H13	119.5
C15—C14—H14	120	C21—C22—C23	119.6 (5)
C13—C14—H14	120	C21—C22—H22	120.2
C13—C12—C11	120.0 (5)	C23—C22—H22	120.2
			
C11—S1—S2—C21	96.8 (2)	C22—C23—C24—C25	−2.1 (8)
S1—S2—C21—C22	−87.5 (5)	C26—C25—C24—C23	0.7 (8)
S1—S2—C21—C26	96.2 (4)	C14—C15—C16—C11	1.2 (7)
C22—C21—C26—C25	−0.7 (8)	C14—C15—C16—N1	−178.3 (4)
S2—C21—C26—C25	175.7 (4)	C12—C11—C16—C15	−0.3 (7)
C22—C21—C26—N2	177.1 (4)	S1—C11—C16—C15	177.5 (4)
S2—C21—C26—N2	−6.5 (6)	C12—C11—C16—N1	179.1 (4)
C21—C26—C25—C24	0.7 (7)	S1—C11—C16—N1	−3.0 (6)
N2—C26—C25—C24	−177.1 (5)	C11—C12—C13—C14	2.1 (8)
C13—C14—C15—C16	−0.4 (7)	C15—C14—C13—C12	−1.2 (8)
C13—C12—C11—C16	−1.3 (7)	C26—C21—C22—C23	−0.8 (8)
C13—C12—C11—S1	−179.1 (4)	S2—C21—C22—C23	−177.2 (4)
S2—S1—C11—C16	103.3 (4)	C24—C23—C22—C21	2.2 (9)
S2—S1—C11—C12	−78.8 (4)		

### Hydrogen-bond geometry (Å, º)

**Table:**

*D*—H···*A*	*D*—H	H···*A*	*D*···*A*	*D*—H···*A*
N1—H1*A*···O1*W*^i^	0.89	1.83	2.723 (6)	178
N1—H1*B*···Cl2^i^	0.89	2.24	3.108 (4)	166
N1—H1*C*···Cl1^i^	0.89	2.25	3.103 (4)	160
N2—H2*A*···O2*W*^ii^	0.89	1.84	2.727 (6)	177
N2—H2*B*···Cl2^iii^	0.89	2.26	3.111 (4)	160
N2—H2*C*···Cl2	0.89	2.30	3.157 (4)	163
O2*W*—H4*W*···Cl2	0.86 (5)	2.36 (5)	3.157 (5)	155 (5)
O2*W*—H3*W*···Cl1^iv^	0.85 (5)	2.23 (5)	3.078 (4)	171 (6)
O1*W*—H1*W*···Cl1^v^	0.85 (5)	2.27 (5)	3.096 (5)	167 (5)
O1*W*—H2*W*···Cl1^iv^	0.86 (5)	2.27 (5)	3.127 (5)	176 (7)

Symmetry codes: (i) −*x*+2, −*y*, *z*+1/2; (ii) *x*, *y*, *z*−1; (iii) −*x*+2, −*y*, *z*−1/2; (iv) *x*, *y*, *z*+1; (v) −*x*+2, −*y*+1, *z*+1/2.
